# Supplementary material for: On the Prognosis of Multifocal Glioblastoma: An Evaluation Incorporating Volumetric MRI
Source: Curr Oncol. 2021 Apr 7;28(2):1437–46. doi: 10.3390/curroncol28020136 (PMC8167648; doi:10.3390/curroncol28020136)
Supplement: Supplementary file 1 [file curroncol-28-00136-s001.pdf]

Table S1. Cox regression with continuous, volumetric parameters. Bold values indicate statistical significance. IV: initial tumor volume; CEV-: non-enhancing tumor area; CEV-/IC: ratio of CEV- to IV; CI: 95 % confidence interval; HR: hazard ratio; RV: residual tumor volume; EOR: extent of resection.

	all			unifocal			multifocal		
	**HR**	**CI**	***p*-value**	**HR**	**CI**	***p*-value**	**HR**	**CI**	***p*-value**
IV	1	0.99–1.01	0.855	1	0.99–1.01	0.943	1	0.99–1.0	0.737
CEV-	1	0.98–1.01	0.204	1	0.98–1.02	0.933	0.97	0.93–1.0	0.07
CEV-/IV	0.989	0.98–1	0.05	1	0.99–1.01	0.793	0.975	0.95–0.99	**0.023**
RV	1.027	1.02–1.04	**< 0.0001**	1.041	1.01–1.07	**0.003**	1.008	1.0–1.02	0.342
EOR	0.98	0.98–0.99	**< 0.0001**	0.99	0.98–0.996	**0.002**	0.99	0.98–0.998	**0.019**

Table S2. Cutoff values calculated by ROC analysis and Younden-Index to categorize statistically significant volumetric parameters. AUC: area under the curve; CEV-: non-enhancing tumor area; CEV-/IV: ratio of CEV- to IV; IV: initial tumor volume; ROC: receiver operating characteristics; RV: residual tumor volume; EOR: extent of resection.

	all		unifocal		multifocal	
	**cutoff**	**AUC**	**cutoff**	**AUC**	**cutoff**	**AUC**
CEV-/IV	-	-	-	-	0.23	0.716
RV	1.04 mL	0.687	0.41 mL	0.634	-	-
EOR	82.5 %	0.703	91.0 %	0.66	27.7 %	0.774

Table S3. Cox regression of volumetric parameters categorized according to thresholds calculated via ROC analysis. Bold values indicate statistical significance. CEV-: non-enhancing tumor area; CEV-/IV: ratio of CEV- to IV; CI: 95 % confidence interval; HR: hazard ratio; IV: initial tumor volume; ROC: receiver operating characteristics; RV: residual tumor volume; EOR: extent of resection.

	all			unifocal			multifocal		
	**HR**	**CI**	***p*-value**	**HR**	**CI**	***p*-value**	**HR**	**CI**	***p*-value**
CEV-/IV	-	-	-	-	-	-	2.2	1.1–4.4	**0.026**
RV	0.46	0.3–0.72	**0.001**	0.6	0.35–1.01	0.05	-	-	-
EOR	0.36	0.24–0.54	**< 0.0001**	0.56	0.35–0.94	**0.027**	0.47	0.26–0.99	**0.047**

Table S4. Kaplan Meier analysis of volumetric parameters categorized according to thresholds calculated via ROC analysis. Comparison of 1-year survival (all, unifocal) and 6-months survival (multifocal), respectively. Survival rates are presented in percent with standard deviation. Bold values indicate statistical significance. IV: initial tumor volume; CEV-: non-enhancing tumor area; CEV-/IV: ratio of CEV- to IV; ROC: receiver operating characteristics; RV: residual tumor volume; EOR: extent of resection.

	all		unifocal		multifocal	
	**1-year survival**	***p*-value**	**1-year survival**	***p*-value**	**6-months survival**	***p*-value**
CEV-/IV	-	-	-	-	62.5 ± 12.1<br>35.5 ± 9.1	**0.023**
RV	77.1 ± 6.1<br>40.8 ± 0.5	**< 0.0001**	82.6 ± 6.5<br>56.0 ± 6.2	0.055	-	-
EOR	72.5 ± 5.3<br>30.6 ± 6.0	**< 0.0001**	73.6 ± 5.7<br>51.6 ± 8.1	**0.025**	66.1 ± 10.8<br>21.3 ± 10.5	**0.043**

Table S5. Cox regression of continuous clinical and pathological parameters. Bold values indicate statistical significance. CI: 95% confidence interval; HR: hazard ratio; MGMT: O6-methylguanine-DNA methyltransferase promoter methylation; MRC-NPS: medical research council neurological performance scale.

	all			unifocal			multifocal		
	**HR**	**CI**	***p*-value**	**HR**	**CI**	***p*-value**	**HR**	**CI**	***p*-value**
age	1.03	1.01–1.05	**0.001**	1.04	1.01–1.07	**0.002**	1.01	0.98–1.04	0.602

MGMT	0.99	0.98–0.996	**0.007**	0.98	0.96–0.99	**0.001**	1.01	1.0–1,02	0.294
MRC-NPS	1.3	1.04–1.64	**0.02**	1.32	0.99–1.76	0.061	1.2	0.8–1.7	0.443
adjuvant therapy	0.28	0.21–0.37	**< 0.0001**	0.24	0.16–0.37	**< 0.0001**	0.38	0.25–0.58	**< 0.0001**
